# Comparison of PET/CT-based eligibility according to VISION and TheraP trial criteria in end-stage prostate cancer patients undergoing radioligand therapy

**DOI:** 10.1007/s12149-023-01874-5

**Published:** 2023-10-27

**Authors:** Kerstin Michalski, Aleksander Kosmala, Rudolf A. Werner, Sebastian E. Serfling, Anna K. Seitz, Constantin Lapa, Andreas K. Buck, Philipp E. Hartrampf

**Affiliations:** 1 Department of Nuclear Medicine, University Hospital Würzburg, Oberdürrbacher Straße 6, 97080 Würzburg, Germany; 2 Department of Urology and Paediatric Urology, University Hospital Würzburg, Oberdürrbacher Straße 6, 97080 Würzburg, Germany; 3 Nuclear Medicine, Medical Faculty, University of Augsburg, Stenglinstr. 2, 86156 Augsburg, Germany

**Keywords:** PSMA radioligand therapy, PET-based eligibility, Dual tracer imaging, VISION, TheraP

## Abstract

**Background:**

Two randomized clinical trials demonstrated the efficacy of prostate-specific membrane antigen (PSMA) radioligand therapy (PSMA RLT) in metastatic castration-resistant prostate cancer (mCRPC). While the VISION trial used criteria within PSMA PET/CT for inclusion, the TheraP trial used dual tracer imaging including FDG PET/CT. Therefore, we investigated whether the application of the VISION criteria leads to a benefit in overall survival (OS) or progression-free survival (PFS) for men with mCRPC after PSMA RLT.

**Methods:**

Thirty-five men with mCRPC who had received PSMA RLT as a last-line option and who had undergone pretherapeutic imaging with FDG and [^68^Ga]Ga-PSMA I&T or [^18^F]PSMA-1007 were studied. Therapeutic eligibility was retrospectively evaluated using the VISION and TheraP study criteria.

**Results:**

26 of 35 (74%) treated patients fulfilled the VISION criteria (= VISION+) and only 17 of 35 (49%) fulfilled the TheraP criteria (= TheraP+). Significantly reduced OS and PFS after PSMA RLT was observed in patients rated VISION− compared to VISION+ (OS: VISION−: 3 vs. VISION+: 12 months, hazard ratio (HR) 3.1, 95% confidence interval (CI) 1.0–9.1, *p* < 0.01; PFS: VISION−: 1 vs. VISION+: 5 months, HR 2.7, 95% CI 1.0–7.8, *p* < 0.01). For patients rated TheraP−, no significant difference in OS but in PFS was observed compared to TheraP+ patients (OS: TheraP−: 5.5 vs. TheraP+: 11 months, HR 1.6, 95% CI 0.8–3.3, *p* = 0.2; PFS: TheraP−: 1 vs. TheraP+: 6 months, HR 2.2, 95% CI 1.0–4.5, *p* < 0.01).

**Conclusion:**

Retrospective application of the inclusion criteria of the VISION study leads to a benefit in OS and PFS after PSMA RL, whereas TheraP criteria appear to be too strict in patients with end-stage prostate cancer. Thus, performing PSMA PET/CT including a contrast-enhanced CT as proposed in the VISION trial might be sufficient for treatment eligibility of end-stage prostate cancer patients.

**Supplementary Information:**

The online version contains supplementary material available at 10.1007/s12149-023-01874-5.

## Introduction

Prostate-specific membrane antigen (PSMA)-directed radioligand therapy showed convincing results in the latest randomized clinical trials (VISION [1] and TheraP trial [2]). The prolongation of survival compared to standard of care shown in the VISION trial [1] led to the approval of the first therapeutic PSMA-directed agent [^177^Lu]Lu-PSMA-617 by the U.S. Food and Drug Administration [3] and the European Medicines Agency [4]. However, there is still no consensus about the PET-based eligibility criteria before PSMA radioligand therapy (RLT) [5]. An adequate PSMA expression on PSMA positron emission tomography (PET)/computed tomography (CT) is recommended [6] but not defined. The VISION study assessed adequate PSMA expression as a tracer uptake visually greater than the liver on [^68^Ga]Ga-PSMA-11 PET/CT [1]. In contrast, the inclusion criteria of the TheraP trial, which compared [^177^Lu]Lu-PSMA-617 with cabazitaxel, referred to the maximum standardized uptake value (SUV_max_) of the tumor lesions and included a second PET/CT scan using [^18^F]Fluorodeoxyglucose (FDG). Patients with lesions showing increased FDG-uptake but no relevant uptake on PSMA PET (FDG+/PSMA−) were excluded from the TheraP trial. Of note, it has been previously shown that for patients with discordant FDG+/PSMA− lesions, who were excluded from the single-arm phase 2 study LuPSMA [7], a poor survival under different alternative treatment options resulted [8]. In a retrospective analysis of our own study group we have shown that FDG+/PSMA− lesions are negative prognostic biomarker in patients undergoing PSMA RLT [9]. The use of a dual tracer PET/CT staging before PSMA RLT is still discussed controversial. An additional FDG PET scan can help to detect aggressive sites of disease [10–12] and lesions which cannot be treated with PSMA RLT, but is still time-consuming, costly and puts further burden on end-stage disease patients [13].

The aim of this retrospective study is to evaluate the PET-eligibility criteria of the VISION and the TheraP trial and to assess their prognostic impact on overall survival (OS) and progression-free-survival (PFS) of patients who were treated with PSMA RLT.

## Materials and methods

### Patient cohort

In this single-center study, 35 patients with mCRPC who had undergone PSMA and FDG PET/CT between June 2018 and January 2020 prior to PSMA RLT with [^177^Lu]Lu-PSMA I&T were included. All patients signed written informed consent. The local Ethics Committee waived the need for further approval due to the retrospective character of this investigation (waiver no. 20190815 01). Parts of this cohort have been reported in [14]. However, that previous analysis did not focus on eligibility criteria for PSMA RLT.

### Imaging and treatment protocol

Whole-body PET scans were acquired as described before [14]. In short, PET/CT were performed either with full-dose contrast-enhanced diagnostic CT (PSMA ligand) or low-dose CT (FDG) for attenuation correction and anatomical co-registration. Both PET/CT studies were performed on two separate days with a median of 25 (1–137) days in between. 23 patients were staged with [^68^Ga]Ga-PSMA I&T and 12 patients were staged with [^18^F]PSMA-1007. Biodistribution of [^68^Ga]Ga-PSMA I&T is comparable to [^68^Ga]Ga-PSMA-11, whereas [^18^F]PSMA-1007 shows a liver-dominant excretion with a higher physiological liver uptake [15]. Standardized institutional protocols for RLT work-up were applied. Radiosynthesis of radiotracers is described elsewhere [14, 16]. The standard PSMA RLT protocol consisted of infusion of 6.0 GBq of the radioligand every 6–8 weeks with up to 4 cycles depending on response to treatment [14].

### Image analysis

PET/CT images were retrospectively analyzed by one nuclear medicine specialist (K.M.) using syngo.via (Siemens Healthcare GmbH, Erlangen, Germany). PSMA PET scans were read blinded to the FDG PET scan according to the VISION criteria: at least one tumor lesion had to present a tracer uptake visually above the physiological tracer uptake of the liver ([^68^Ga]Ga-PSMA I&T) or the spleen ([^18^F]PSMA-1007, according to [15, 17, 18]) as a reference organ. Patients were excluded in case of PSMA-negative (tracer uptake equal or less than the liver/spleen) metastases if they measured at least 1.0 cm (bone metastases with soft tissue component), 1.0 cm (visceral metastases) or 2.5 cm (lymph nodes in short axis) [1]. For the inclusion according to the TheraP criteria, patients had to present a SUV_max_ ≥ 20 at a site of disease and a SUV_max_ ≥ 10 at all sites of measurable disease (≥ 1.0 cm) on PSMA PET/CT. Patients were excluded in case of discordant FDG+/PSMA− tumor lesions [2]. These were defined as metastases with a tracer uptake greater than the liver on FDG PET but less or equal than the liver/spleen on PSMA PET.

### Statistical analysis

For statistical analyses, we used GraphPad Prism version 9.3.0 (GraphPad Software, San Diego, California, United States). Unless otherwise described data are presented in median and range in parentheses. The time interval between the day of the first RLT and day of death was defined as OS (presented as median). The time interval between imaging-based progression on PSMA PET/CT [according to RECIP 1.0 criteria [19], assessed by one reader (K.M.)] or progression of prostate-specific antigen (PSA) according to PCWG3-criteria [20] or death before first re-staging was defined as PFS (presented as median). We used Kaplan–Meier curves and log-rank comparison for calculation and comparison of OS and PFS between eligible and non-eligible patients. Uni- and multivariable analysis was undertaken for stratification of probable prognostic markers for OS. *p* < 0.05 was considered statistically significant.

## Results

### Patient characteristics

35 PCa patients with a median age of 73 (46–90) years and PSMA and FDG PET/CT prior to PSMA RLT were included in the final analysis. In median, 70 (17–313) months lay between first diagnosis of PCa and initiation of RLT. Patients with a median initial Gleason score of 8 (5–10) were treated with a median of three (1–9) cycles with a median cumulative activity of 18.3 (4.9–54.8) GBq [^177^Lu]Lu-PSMA I&T. The median OS in the entire cohort was 9 months and the median PFS 3 months. Detailed characteristics can be found in Table 1.

**Table 1:** Patient characteristics

	All patients (*n* = 35)
Age (years)	73.0 (46–90)
Time since diagnosis of prostate cancer (months)	70 (17–313)
Gleason score	8 (5–10)
PSA (ng/ml)	157 (0.07–5000)
ECOG	0–2
Sites of disease	*n* (patients)
Prostate/local	13
Lymph node	17
Bone	35
Liver	10
Lung	4
Other	4
Previous treatment	*n* (patients)
Prostatectomy	20
Radiotherapy to prostate/prostate bed	15
ADT	34^a^
Abiraterone	28
Enzalutamide	27
Docetaxel	24
Cabazitaxel	10
Median lines of treatment before RLT	3 (2–5)
Number of RLT cycles	*n* (patients)
1 cycle	35
2 cycles	25
3 cycles	21
4 cycles	15
4 > cycles	5

^a^Indicates one patient with orchiectomy

### Eligibility for PSMA RLT according to VISION and TheraP criteria

**Fig. 1:**
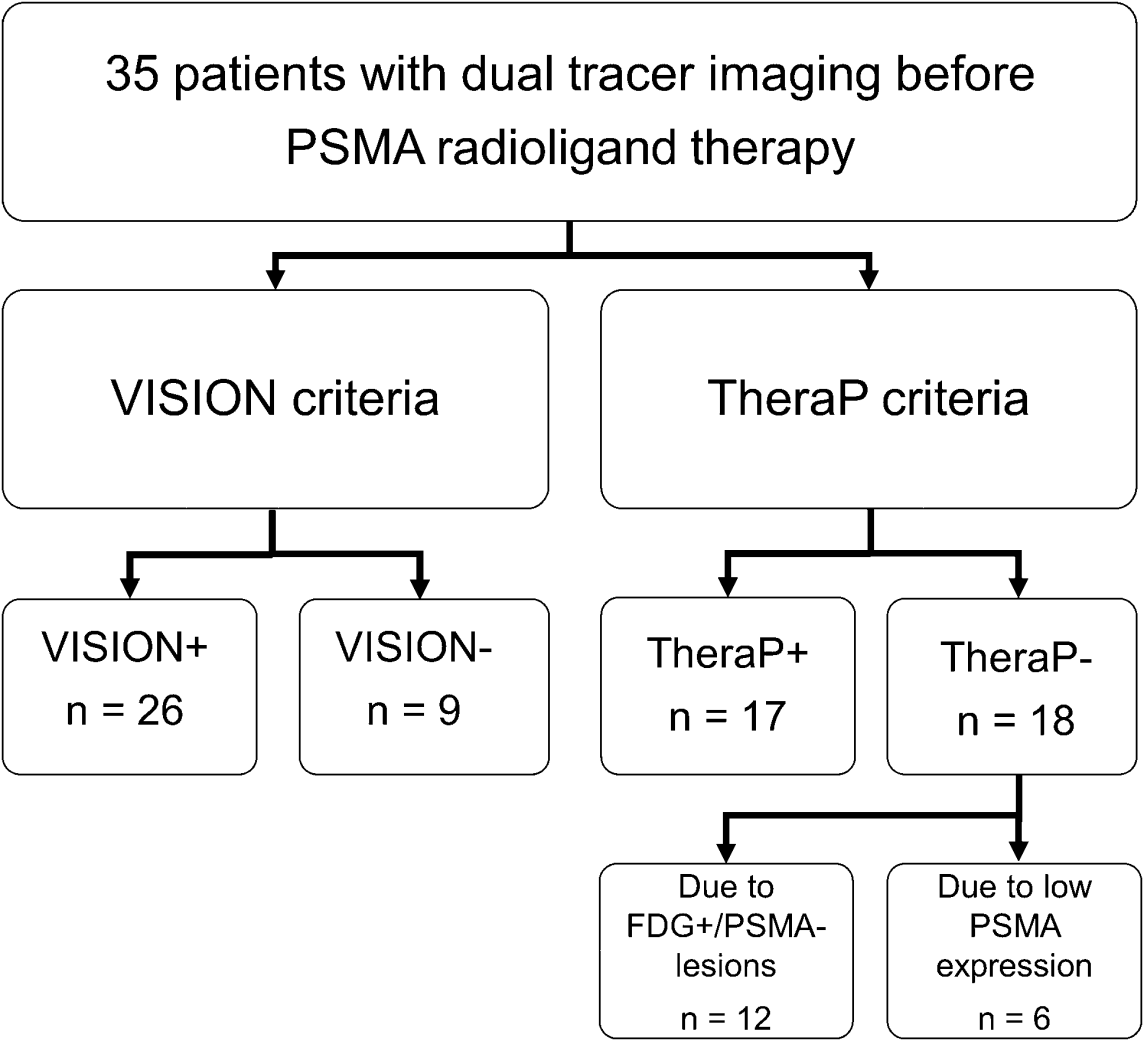
Flowchart of retrospective application of the selection criteria used in the VISION and the TheraP trial

**Fig. 2:**
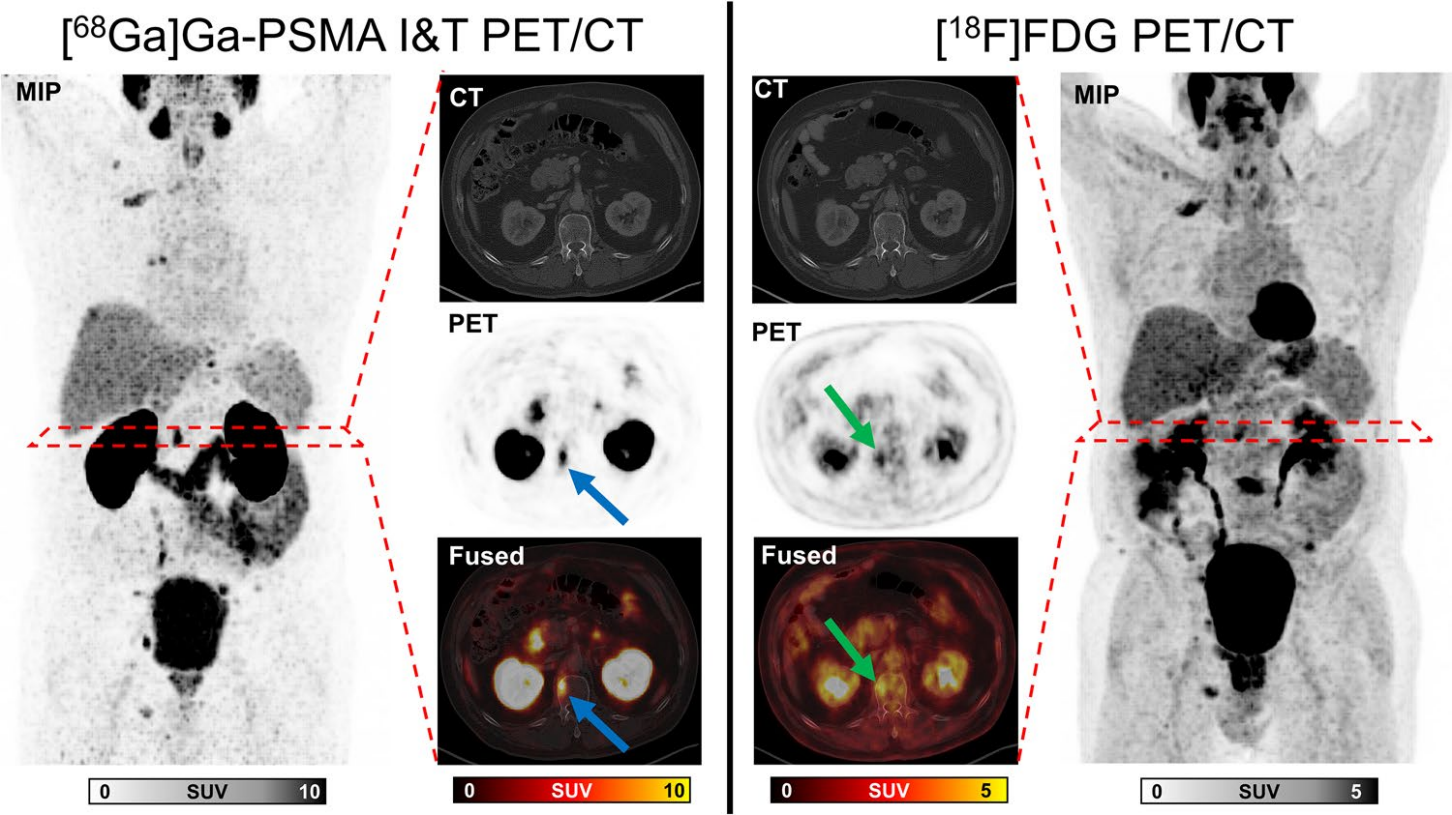
Maximum intensity projections (MIP), CT, PET, and fused images of a man eligible according to VISION criteria (VISION+) but excluded due to TheraP criteria (TheraP−). MIP of [^68^Ga]Ga-PSMA I&T PET (left column) demonstrated multiple lymph node and bone metastases. Highest maximum standardized uptake value (SUV_max_) on [^68^Ga]Ga-PSMA I&T PET was found in the first lumbal vertebral body (blue arrows; SUV_max_ 17.5). The metastasis showed a high uptake (green arrow; SUV_max_ 6.3) on FDG PET (right column). No FDG-positive/PSMA-negative lesions were detected. The patient did not meet the TheraP criteria because no tumor lesions had a SUV_max_ ≥ 20. The patient survived 12 months after initiation of PSMA radioligand therapy

**Fig. 3:**
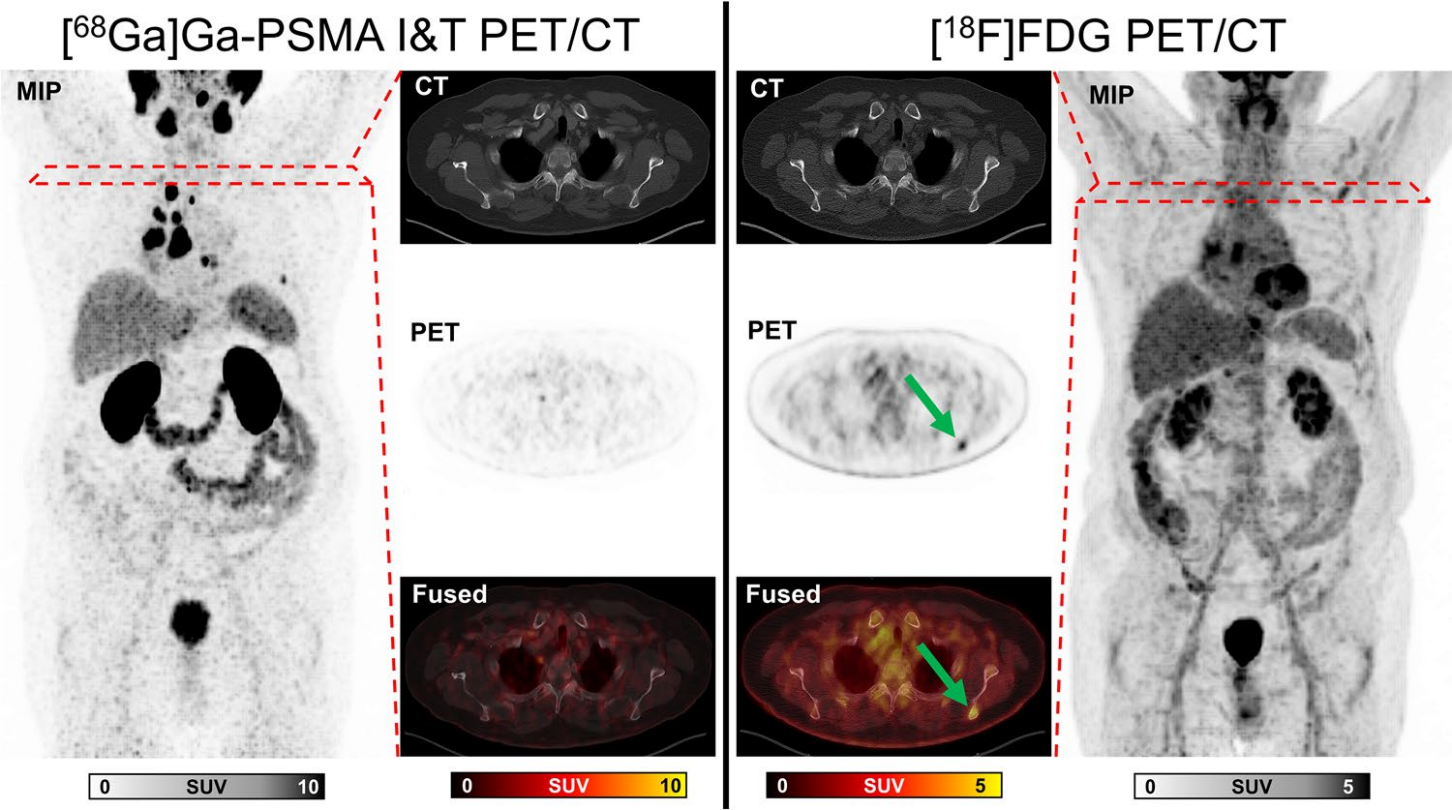
Maximum intensity projections (MIP), CT, PET, and fused images of a man eligible according to VISION criteria (VISION+) but excluded due to TheraP criteria (TheraP−) because of a FDG+ /PSMA− bone metastasis in the left scapula. MIP of [^68^Ga]Ga-PSMA I&T PET (left column) demonstrated bone and lymph node metastases. MIP of FDG PET (right column) showed an additional metastasis in the left scapula (green arrow, SUVmax 5,0), which was not seen on [^68^Ga]Ga-PSMA I&T PET. The metastasis was confirmed on follow-up imaging showing increasing osteoblastic reaction. The patient survived 7 months after initiation of PSMA radioligand therapy

26 of 35 (74%) treated patients fulfilled the VISION criteria (= VISION+) and only 17 of 35 (49%) fulfilled the TheraP criteria (= TheraP+). Regarding patients rated TheraP−, discordant FDG-positive/PSMA-negative (= FDG+/PSMA−) tumor lesions were found in 12 of 18 patients. Another 6 of 18 patients were retrospectively excluded due to low PSMA expression: 4 patients did not have a SUV_max_ of ≥ 20 at any site and ≥ 10 in other measureable sites of disease. One patient was excluded either due to a missing SUV_max_ of ≥ 20 in one lesion or due to a SUV_max_ < 10 in other lesions, respectively (Fig. 1). All patients who would have been excluded according to VISION trial criteria (*n* = 9) were also rated TheraP− because of FDG+/PSMA− metastases. All patients who would have been eligible under the TheraP criteria would also have been eligible under the VISION criteria. Nine patients were eligible based on VISION but not TheraP criteria (Fig. 2). FDG+/PSMA− sites of disease (in lymph nodes and bone) were found in 3 of 26 (12%) patients rated VISION+ (Fig. 3).

### Reduced OS and PFS in patients who retrospectively would not have been eligible for PSMA RLT

**Fig. 4:**
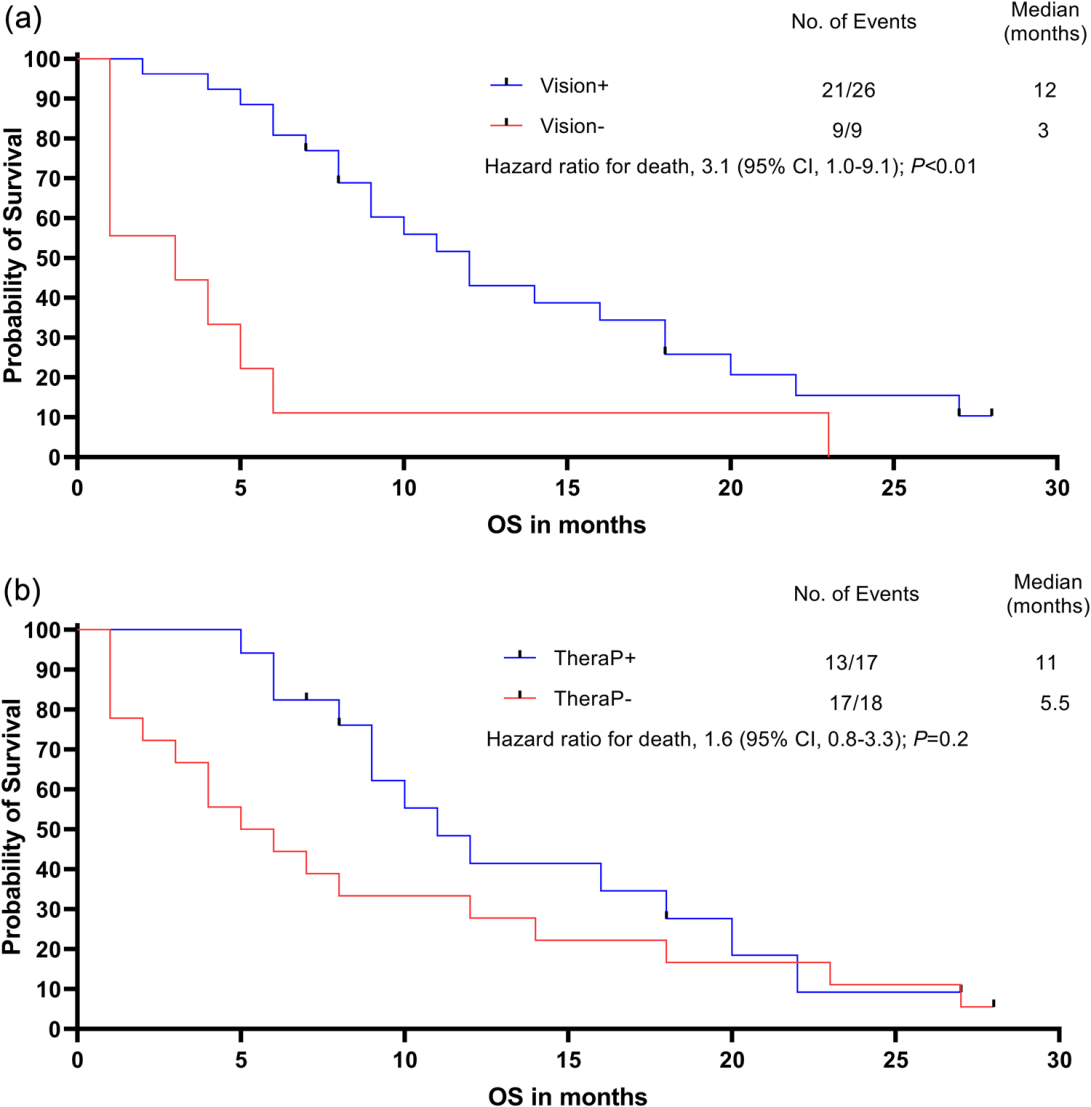
Kaplan–Meier curves of median overall survival. **a** Patients who would have been excluded according to VISION criteria (VISION−; red line) showed significantly reduced overall survival after PSMA radioligand therapy (3 vs. 12 months, HR 3.1, 95% CI 1.0–9.1, *p* < 0.01). **b** Patients who would have been excluded according to TheraP criteria (TheraP−, red line) had no significant difference in survival after PSMA radioligand therapy (5.5 vs. 11 months, HR 1.6, 95% CI 0.8–3.3, *p* = 0.2)

**Fig. 5:**
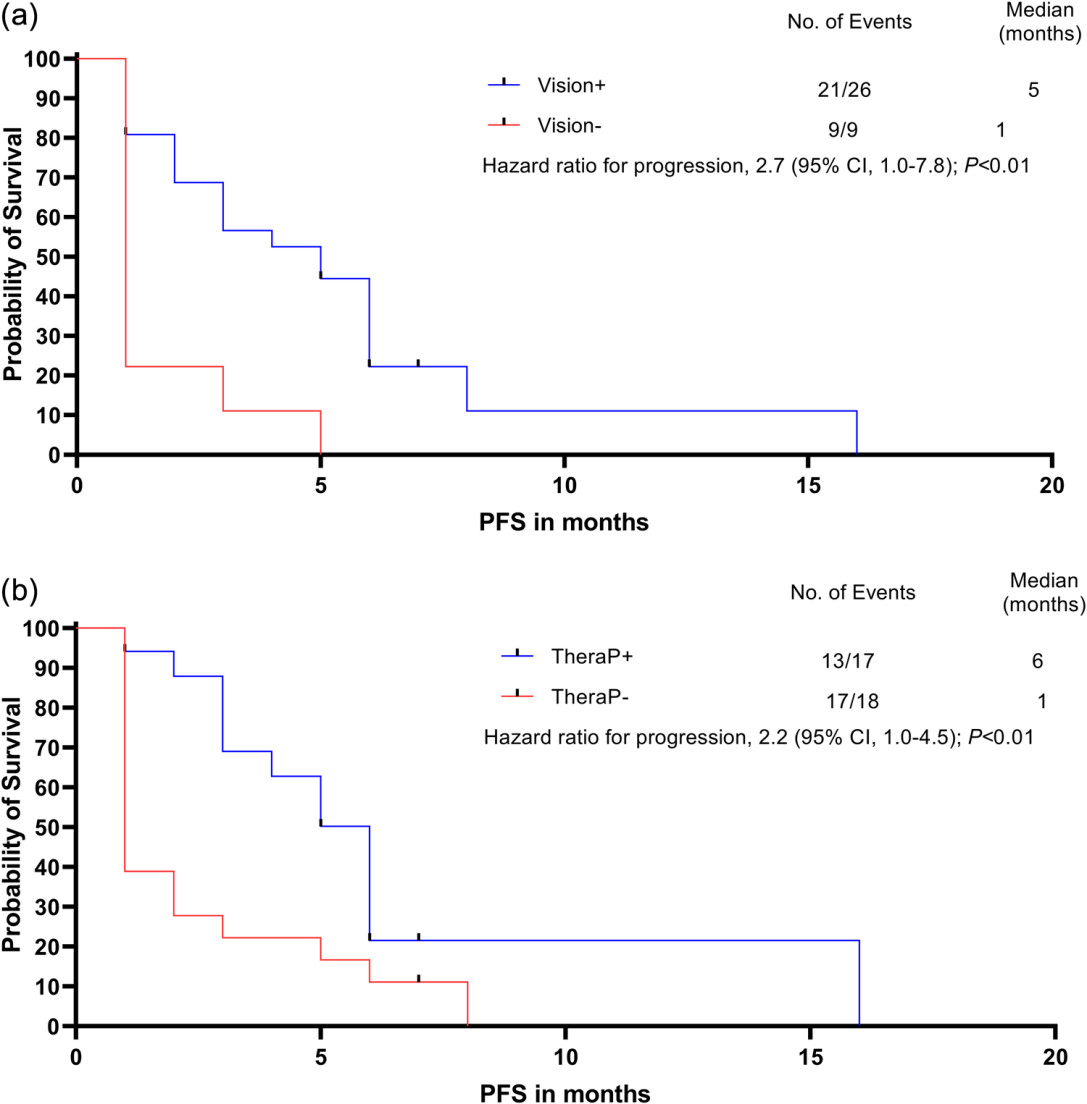
Kaplan–Meier curves of progression-free survival (PFS). **a** Patients who would have been excluded according to VISION criteria (VISION−; red line) showed significantly reduced PFS after PSMA radioligand therapy (1 vs. 5 months, HR 2.7, 95% CI 1.0–7.8, *p* < 0.01). **b** Patients who would have been excluded according to TheraP criteria (TheraP−, red line) had also a significant difference in PFS after PSMA radioligand therapy (1 vs. 6 months, HR 2.2, 95% CI 1.0–4.5, *p* < 0.01)

Patients who would have been excluded according to VISION criteria showed significantly reduced OS after PSMA RLT (VISION−: 3 vs. VISION+: 12 months, HR 3.1, 95% CI 1.0–9.1, *p* < 0.01, Fig. 4a). For patients who would have been excluded according to TheraP criteria difference in survival after PSMA RLT was not significant (TheraP−: 5.5 vs. TheraP: 11 months, HR 1.6, 95% CI 0.8–3.3, *p* = 0.2, Fig. 4b). In analogy, PFS was also significantly reduced in patients rated VISION− (VISION−: 1 vs. VISION+: 5 months, HR 2.7, 95% CI 1.0–7.8, *p* < 0.01, Fig. 5a). Furthermore, PFS was also significantly shortened in patients rated TheraP− (TheraP−: 1 vs. TheraP+: 6 months, HR 2.2, 95% CI 1.0–4.5, *p* < 0.01, Fig. 5b).

Within patients rated TheraP−, patients excluded due to FDG+/PSMA− lesions showed significantly shorter median OS of 4.5 months (HR 2.5, 95% CI 1.0–6.2, *p* = 0.01) compared to TheraP+ patients with 11 months. No significant difference in median OS was found for patients with low PSMA expression with 15 months (HR 0.8, 95% CI 0.3–2.1, *p* = 0.6) compared to TheraP+ patients. Combining VISION and TheraP criteria resulted in four different categories (Table 2). Patients rated VISION+/TheraP+ showed longest survival of 11 months compared to patients rated VISION−/TheraP− with 3 months (HR 2.8, 95% CI 1.0–7.9, *p* < 0.01). No significant difference in OS was found compared to patients rated VISION+/TheraP− with 12 months (HR 1.0, 95% CI 0.4–2.5, *p* = 0.92 (Supplemental Fig. S1).

**Table 2:** Rating of *n* = 35 patients according to the VISION and TheraP trial criteria

	TheraP+	TheraP−	Total
VISION+	17	9	26
VISION−	0	9	9
Total	17	18	35

The same results were found for PFS: within patients rated TheraP−, patients excluded due to FDG+/PSMA− lesions showed significantly shorter median PFS of 1 month (HR 3.3, 95% CI 1.2–8.4, *p* < 0.001) compared to TheraP+ patients with 6 months. No significant difference in median PFS was found for patients with low PSMA expression with 4 months (HR 1.2, 95% CI 0.4–3.4, *p* = 0.7) compared to TheraP+ patients. Patients rated VISION+/TheraP+ showed longest PFS of 6 months compared to patients rated VISION−/TheraP− with 1 month (HR 0.3, 95% CI 0.1–0.9, *p* < 0.001). No significant difference in PFS was found compared to patients rated VISION+/TheraP− with 2 months (HR 0.6, 95% CI 0.2–1.6, *p* = 0.2; Supplemental Fig. S2).

On univariable analysis, only eligibility according to VISION criteria (HR 0.28, 95% CI 0.13–0.68, *p* = 0.003) was significantly associated with OS. Multivariable Cox regression analysis adjusting for PSA values, Gleason Score and previous chemotherapy with cabazitaxel then confirmed that eligibility according to VISION criteria (HR 0.28, 95% CI 0.12–0.71, *p* = 0.005) is significantly associated with longer overall survival (Table 3).

**Table 3:** Univariable and multivariable analysis of VISION and TheraP-eligibility

	Univariable analysis			Multivariable analysis		
	HR	95% CI	*p*-value	HR	95% CI	*p*-value
PSA µg/l	1.00	1.00–1.00	0.20	1.00	1.00–1.00	0.08
Gleason score	0.69	0.47–1.02	0.06	0.70	0.45–1.09	0.11
Chemotherapy with Cabazitaxel	1.18	0.49–2.56	0.69	1.18	0.45–2.81	0.72
VISION eligible (yes)	0.28	0.13–0.68	0.003	0.28	0.12–0.71	0.005
TheraP eligible (yes)	0.60	0.28–1.24	0.17			

## Discussion

This study included 35 patients who underwent dual-tracer imaging with PSMA and FDG PET/CT prior to initiation of PSMA RLT. This cohort allowed retrospectively evaluating the different eligibility criteria of the VISION trial and the TheraP trial and their impact on clinical outcome in a patient cohort with end-stage prostate cancer. OS and PFS was significantly longer in patients eligible for treatment by VISION criteria than in patients ineligible for treatment, whereas TheraP criteria were only significantly associated with PFS but not OS. This could be due to the definition of low PSMA expression used in the TheraP trial or the patients characteristics in our patient cohort, which differ from the patients included in the TheraP trial: while the TheraP trial included patients treatment-naïve to cabazitaxel, parts of the patients in our cohort (29%) and the patients included in the VISION trial (approx. 42%) were pretreated with cabazitaxel. In this regard, it is not surprising that our patient cohort had a relatively short median OS of only 9 months compared to the PSMA RLT arm in the VISION trial with 15 months. This is probably due to “selection bias” of patients to be included in a randomized clinical trial in good clinical conditions and patients who were treated in our department on compassionate use basis as a last line option.

FDG+/PSMA− sites of disease represent a negative prognostic biomarker in patients with mCRPC under standard treatments. Patients excluded from the LuPSMA trial with discordant FDG+/PSMA− lesions showed a median OS of only 3.9 months [8]. In our own analysis (also including patients evaluated in the present study), patients with FDG+/PSMA− sites of disease had a reduced median OS of 6.0 months compared to 16.0 months under PSMA RLT [9]. Tumor heterogeneity is a frequent phenomenon in prostate cancer. Fourquet et al. revealed a low concordance between [^18^F]F-DCFPyL and FDG PET/CT imaging of only 22% in a prospective study [21]. A high glucose metabolism is a surrogate parameter of a more aggressive disease with less response to therapy, independent of the treatment. A high metabolic tumor volume on FDG PET was a negative prognostic marker for PSA-response before PSMA RLT and cabazitaxel in the TheraP trial [22]. Hence, there is no doubt that an additional FDG PET is helpful in the detection of PSMA-negative, more aggressive tumor lesions, which could reduce response to PSMA RLT. FDG PET could, therefore, possibly identify patients who don’t benefit of PSMA RLT, which might also help saving costs in the end. Seifert et al. found in a retrospective VISION-like analysis a minor fraction of only 3 of 89 patients who were considered VISION+ but showed FDG+/PSMA− sites of tumor in a patient cohort screened for PSMA RLT. In our smaller study group of 35 patients treated with PSMA RLT, 3 of 26 patients rated VISION+ had discordant FDG+/PSMA− lesions. Still, the application of the VISION criteria was a significant prognosticator for patient outcome in an end-stage setting and might therefore be sufficient for patient selection in this setting.

The use of TheraP selection criteria did not show a significant difference in OS but in PFS. This could be due to our small sample size and the different pre-treatments in our patient cohort. Furthermore, patients who were regarded to have a low or insufficient PSMA expression according to TheraP trial criteria were evaluated to have adequate PSMA expression in our clinical routine and were, therefore, treated with PSMA RLT. In this sense, our cohort is not exactly comparable to the patients treated in the TheraP trial and the TheraP criteria appear to be too strict to apply in patients with end-stage prostate cancer. A high tracer uptake on PSMA PET was significantly associated with a better outcome after PSMA RLT both in the VISION [23] and the TheraP [22] trial, whereas low PSMA expression is a negative prognostic marker [24]. The inclusion of only patients with a very high PSMA expression represents a selection of patients with promising outcome, but might exclude patients who would have responded to therapy.

Our study has some limitations. The retrospective character of this study results in a small patient cohort. However, this is to our knowledge the only cohort of patients, which evaluates pretherapeutic dual tracer staging in patients who underwent PSMA RLT. Patients evaluated in this study were deemed eligible to PSMA RLT in case of a visual satisfactory assessment of PSMA expression and missing therapeutic alternatives despite discordant lesions on FDG PET. In this sense, such a patient cohort is rare and allows to evaluate retrospectively the inclusion criteria of the prospective clinical trials. Of note, our results use the eligibility criteria of the TheraP and the VISION trial, but our patient cohort is not equal to the patient cohorts treated in these clinical trials and does hence not give suggestions in patients before treatment with cabazitaxel. We evaluated PSMA PET scans using [^68^Ga]Ga-PSMA I&T and [^18^F]PSMA-1007 compared to the radiotracer [^68^Ga]Ga-PSMA-11 used in the VISION and TheraP trial. Especially regarding the criteria used in the TheraP trial, demanding a defined minimum SUVmax, this might have influenced our results. However, this reflects the clinical routine with a lot of different radiopharmaceuticals. Because of the hepatobiliary excretion of [^18^F]PSMA-1007 [25] we used the spleen as a visual reference organ [15, 17, 18]. Similarly, we used [^177^Lu]Lu-PSMA I&T and not [^177^Lu]Lu-PSMA-617 for PSMA RLT, but both radiopharmaceuticals are considered comparable in their efficacy [6, 26].

## Conclusions

Retrospective application of the inclusion criteria of the VISION study leads to a benefit in OS and PFS after PSMA RL, whereas TheraP criteria appear to be too strict in patients with end-stage prostate cancer. Thus, performing PSMA PET/CT including a contrast-enhanced CT as proposed in the VISION trial might be sufficient for treatment eligibility of end-stage prostate cancer patients.

### Supplementary Information

Below is the link to the electronic supplementary material.

Supplementary file1 (TIF 1387 KB). Supplemental Figure 1: Kaplan-Meier curves of median overall survival. **a**) Subgrouping of patients according to the reason of TheraP criteria. Patients excluded due to FDG+/PSMA− lesions showed significantly shorter median OS of 4.5 months (HR 2.5, 95% CI 1.0–6.2, *p* = 0.01; red line) compared to TheraP+ patients with 11 months (green line). No significant difference in median OS was found for patients with low PSMA expression with 15 months (HR 0.8, 95% CI 0.3–2.1, *p* = 0.6; blue line) compared to TheraP+ patients. **b**) Subgrouping of patients according to their VISION and TheraP eligibility. Patients rated VISION+/TheraP+ showed longest survival of 11 months (green line) compared to patients rated VISION−/TheraP− with 3 months (HR 2.8, 95% CI 1.0–7.9, *p* < 0.01; red line). No significant difference in OS was found compared to patients VISION+/TheraP− with 12 months (HR 1.0, 95% CI 0.4–2.5, *p* = 0.92; blue line).

Supplementary file2 (TIF 1376 KB). Supplemental Figure 2: Kaplan–Meier curves of progression-free survival (PFS). **a**) Subgrouping of patients according to the reason of TheraP criteria. Patients excluded due to FDG+/PSMA- lesions showed significantly shorter median PFS of 1 months (HR 3.3, 95% CI 1.2–8.4, *p* < 0.001; red line) compared to TheraP+ patients with 6 months (green line). No significant difference in median OS was found for patients with low PSMA expression with 4 months (HR 1.2, 95% CI 0.4–3.4, *p* = 0.7; blue line) compared to TheraP+ patients. **b**) Subgrouping of patients according to their VISION and TheraP eligibility. Patients rated VISION+/TheraP+ showed longest PFS of 6 months (green line) compared to patients rated VISION−/TheraP− with 1 months (HR 0.3, 95% CI 0.1–0.9, *p* < 0.001; red line). No significant difference in PFS was found compared to patients VISION+/TheraP− with 2 months (HR 0.6, 95% CI 0.2–1.6, *p* = 0.2; blue line).

## Data Availability

Data are available upon reasonable request.
