# Supplementary figures and images for: Comparison of PET/CT-based eligibility according to VISION and TheraP trial criteria in end-stage prostate cancer patients undergoing radioligand therapy
Source: Ann Nucl Med. 2023 Oct 27;38(2):87–95. doi: 10.1007/s12149-023-01874-5 (PMC10822822; doi:10.1007/s12149-023-01874-5)

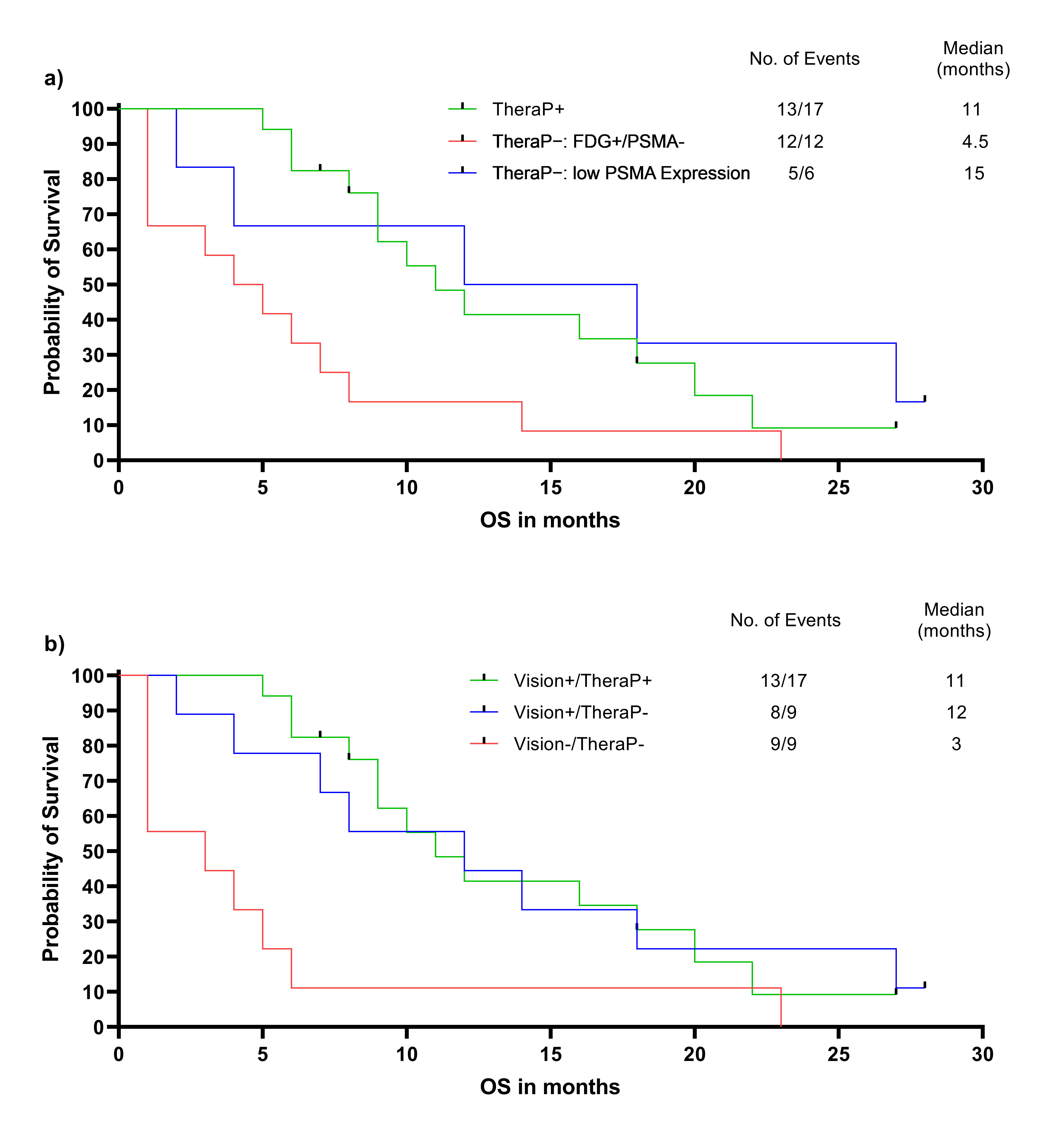

Supplement: Supplementary file 1 — Supplementary file1 (TIF 1387 KB). Supplemental Figure 1: Kaplan-Meier curves of median overall survival. a) Subgrouping of patients according to the reason of TheraP criteria. Patients excluded due to FDG+/PSMA− lesions showed significantly shorter median OS of 4.5 months (HR 2.5, 95% CI 1.0–6.2, p = 0.01; red line) compared to TheraP+ patients with 11 months (green line). No significant difference in median OS was found for patients with low PSMA expression with 15 months (HR 0.8, 95% CI 0.3–2.1, p = 0.6; blue line) compared to TheraP+ patients. b) Subgrouping of patients according to their VISION and TheraP eligibility. Patients rated VISION+/TheraP+ showed longest survival of 11 months (green line) compared to patients rated VISION−/TheraP− with 3 months (HR 2.8, 95% CI 1.0–7.9, p < 0.01; red line). No significant difference in OS was found compared to patients VISION+/TheraP− with 12 months (HR 1.0, 95% CI 0.4–2.5, p = 0.92; blue line). [file 12149_2023_1874_MOESM1_ESM.tif]

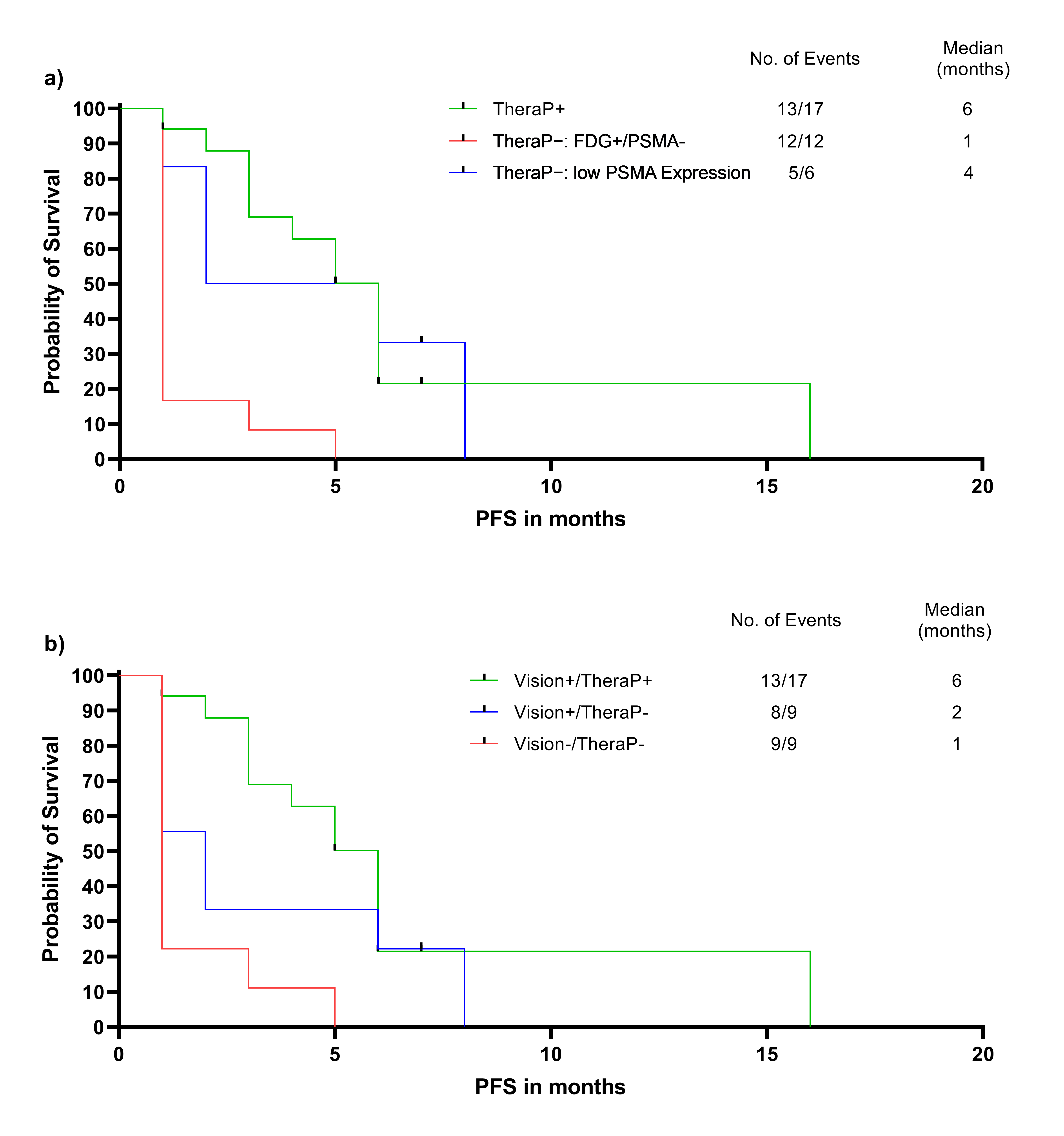

Supplement: Supplementary file 2 — Supplementary file2 (TIF 1376 KB). Supplemental Figure 2: Kaplan–Meier curves of progression-free survival (PFS). a) Subgrouping of patients according to the reason of TheraP criteria. Patients excluded due to FDG+/PSMA- lesions showed significantly shorter median PFS of 1 months (HR 3.3, 95% CI 1.2–8.4, p < 0.001; red line) compared to TheraP+ patients with 6 months (green line). No significant difference in median OS was found for patients with low PSMA expression with 4 months (HR 1.2, 95% CI 0.4–3.4, p = 0.7; blue line) compared to TheraP+ patients. b) Subgrouping of patients according to their VISION and TheraP eligibility. Patients rated VISION+/TheraP+ showed longest PFS of 6 months (green line) compared to patients rated VISION−/TheraP− with 1 months (HR 0.3, 95% CI 0.1–0.9, p < 0.001; red line). No significant difference in PFS was found compared to patients VISION+/TheraP− with 2 months (HR 0.6, 95% CI 0.2–1.6, p = 0.2; blue line). [file 12149_2023_1874_MOESM2_ESM.tif]
